# To Set Up a Logistic Regression Prediction Model for Hepatotoxicity of Chinese Herbal Medicines Based on Traditional Chinese Medicine Theory

**DOI:** 10.1155/2016/7273940

**Published:** 2016-08-29

**Authors:** Hongjie Liu, Tianhao Li, Lingxiu Chen, Sha Zhan, Meilan Pan, Zhiguo Ma, Chenghua Li, Zhe Zhang

**Affiliations:** ^1^Department of Traditional Chinese Medicine, Medical College, Jinan University, Guangzhou 510632, China; ^2^Department of Chinese Herbal Medicines, Pharmacy College, Jinan University, Guangzhou 510632, China; ^3^Biomaterial Laboratory of Guangdong Higher Education and Department of Biomedical Engineering, Jinan University, Guangzhou 510632, China; ^4^Shandong University of Traditional Chinese Medicine, Jinan 250355, China

## Abstract

*Aims*. To establish a logistic regression (LR) prediction model for hepatotoxicity of Chinese herbal medicines (HMs) based on traditional Chinese medicine (TCM) theory and to provide a statistical basis for predicting hepatotoxicity of HMs.* Methods*. The correlations of hepatotoxic and nonhepatotoxic Chinese HMs with four properties, five flavors, and channel tropism were analyzed with chi-square test for two-way unordered categorical data. LR prediction model was established and the accuracy of the prediction by this model was evaluated.* Results*. The hepatotoxic and nonhepatotoxic Chinese HMs were related with four properties (*p* < 0.05), and the coefficient was 0.178 (*p* < 0.05); also they were related with five flavors (*p* < 0.05), and the coefficient was 0.145 (*p* < 0.05); they were not related with channel tropism (*p* > 0.05). There were totally 12 variables from four properties and five flavors for the LR. Four variables, warm and neutral of the four properties and pungent and salty of five flavors, were selected to establish the LR prediction model, with the cutoff value being 0.204.* Conclusions*. Warm and neutral of the four properties and pungent and salty of five flavors were the variables to affect the hepatotoxicity. Based on such results, the established LR prediction model had some predictive power for hepatotoxicity of Chinese HMs.

## 1. Introduction

Chinese herbal medicines (HMs) have been worldwide applied [1], and the concern for their safety is increasing [2]. It is reported that Chinese HMs account for drug-induced liver injury in the second place in China [3]. Many Chinese HMs previously considered to be “nontoxic” have been found to have potential hepatotoxicity [4, 5].

Hepatotoxicity is an important part of Chinese HMs toxicity which is one aspect of* Yao Xin*, the innate nature of Chinese HMs on which Chinese HMs depend to exert or generate the effect.* Yao Xin* includes five aspects: four properties, five flavors, channel tropism, ascending and descending and floating and sinking, and toxicity [6]. Four properties aspect is one of the innate natures to treat cold or warm disease, actually including cold, hot, warm, cool, and neutral, five properties. Five flavors are the real taste of Chinese HMs at first. Gradually the connotation of five flavors changed, and five flavors become one of the innate natures of Chinese HMs, actually including sour, bitter, sweet, pungent, salty, tasteless, and astringent, seven tastes. Channel tropism is the specific action on certain part of human by Chinese HMs. Ascending and descending and floating and sinking aspect is the direction of the effect on human body by Chinese HMs. Toxicity is the injury or damage on human body by Chinese HMs, which could be further divided into hepatotoxicity, nephrotoxicity, cardiotoxicity, and so on.* Yao Xin* of the Chinese HMs is the foundation or basis for the generation of specific function of Chinese HMs [7].

The five aspects of* Yao Xin* are interrelated [8]. Hepatotoxicity, as one aspect of toxicity, is corelated with other four aspects of* Yao Xin*, and the degree of this correlation to what extent has stimulated the interest of many researchers. Since, for most Chinese HMs, ascending and descending and floating and sinking aspect is not mentioned at all, research mainly focuses on relationship between the hepatotoxicity and four properties and five flavors and channel tropism [9, 10].

In this research, we explored the relationship between the hepatotoxicity and four properties and five flavors and channel tropism based on the statistical analysis. The correlation between the independent variables (four properties, five flavors, and channel tropism) and the dependent variable (hepatotoxicity) was analyzed using chi-square test for two-way unordered categorical data, to identify the dependent variables affected by the independent variables. Unconditional logistic regression (LR) was applied to screen the influential factors, and the LR prediction model was established for hepatotoxicity of Chinese HMs. By this way we can predict hepatotoxicity of Chinese HMs based on the combination of TCM theory and modern statistics.

## 2. Materials

### 2.1. Data Sources

*Academic Journals*. The journals include China National Knowledge Infrastructure (CNKI) (1989–2014), VIP Journal Integration Platform (VJIP) (1989–2014), and China Biology Medicine Database (1989–2014).

*Books*. The books include* Drug Induced Liver Disease* [11],* Modern Chinese Herbal Medicines Toxicology* [12],* The Practical Encyclopedia of Acute Poisoning* [13],* Toxic Materia Medica* [14],* Chinese Materia Medica* [2],* Modern Research and Application of Commonly Used Toxic Chinese Herbal Medicines* [15], and* Selected Edition of Chinese Materia Medica* [16].

### 2.2. Data Selected

#### 2.2.1. Selection of Hepatotoxic Chinese HMs

*Inclusion Criteria*. (I) Route of administration is oral administration. (II) Subjects include humans and animals (mice, rats, rabbits, and dogs). (III) The determination of hepatotoxic Chinese HMs include (1) liver damage; (2) clinical manifestations including fever, lassitude, anorexia, rash, itching, jaundice, and uncomfortable feeling or pain in the hepatic area; (3) the clinical signs including hepatomegaly, hepatic congestion, cholestasis, hepatic fibrosis, hepatic cirrhosis, hepatic vasculopathy, hepatic tumor, and liver shrinkage; (4) hepatocyte degeneration or necrosis. Criteria for human hepatotoxicity are (1) plus (2) or (3). Criteria for animal hepatotoxicity are (1) plus (3) or (4) [11, 17].

*Exclusion Criteria*. The description of the Chinese HMs lacks one of the three categories (four properties, five flavors, and channel tropism).

There were 107 hepatotoxic Chinese HMs selected from the data sources based on the above criteria of inclusion and exclusion.

#### 2.2.2. Selection of Nonhepatotoxic Chinese HMs

*Inclusion Criteria*. Chinese HMs in the* Selected Edition of Chinese Materia Medica* [16] were found without hepatotoxicity in the data sources. The definition of hepatotoxicity is described in the part for selection of hepatotoxic Chinese HMs.

*Exclusion Criteria*. The description of the Chinese HMs lacks one of the three categories (four properties, five flavors, and channel tropism).

There were 431 nonhepatotoxic Chinese HMs selected from the data sources based on the above criteria of inclusion and exclusion.

### 2.3. The Method for Processing Data

Four properties of hepatotoxic and nonhepatotoxic Chinese HMs are cold, hot, warm, cool, and neutral, actually 5 kinds; five flavors are sour, bitter, sweet, pungent, salty, tasteless, and astringent, actually 7 kinds; channel tropism involves lung meridian, large intestine meridian, stomach meridian, spleen meridian, heart meridian, small intestine meridian, bladder meridian, kidney meridian, pericardium meridian, triple energizer meridian, gall bladder meridian, and liver meridian, totally 12 kinds. The four properties, five flavors, and channel tropism of the Chinese HMs described in the* Chinese Materia Medica* [2] were set as the standard.

During the data processing, the 24 independent variables of a single Chinese HM (cold, hot, warm, cool, neutral, sour, bitter, sweet, pungent, salty, tasteless, astringent, lung channel, stomach channel, spleen channel, large intestine channel, heart channel, small intestine channel, bladder channel, kidney channel, pericardium channel, triple energizer channel, gall bladder channel, and liver channel) were divided into two categories, “with” and “without”. Category of “with” was coded 1, while category of “without” was coded 0. For example, for Chinese HM* shān yào* (*Rhizoma Dioscoreae*), neutral property, sweet flavor, and entering the lung, spleen, and kidney meridians, the data were processed as follows: cold 0, warm 0, cool 0, neutral 1, sour 0, bitter 0, sweet 1, pungent 0, salty 0, tasteless 0, astringent 0, lung channel 1, stomach channel 0, spleen channel 1, large intestine channel 0, heart channel 0, small intestine 1, bladder channel 0, kidney channel 1, pericardium channel 0, triple energizer 0, gall bladder channel 0, and liver channel 0.

Also, the dependent variable (hepatotoxicity or nonhepatotoxicity of Chinese HMs) is binary which takes values 1 or 0. Hepatotoxicity was coded 1 and nonhepatotoxicity was coded 0. For example, Chinese HM* ài yè* (*Folium Artemisiae Argyi*) with hepatotoxicity was coded 1; another Chinese HM* shān yào* (*Rhizoma Dioscoreae*) without hepatotoxicity was coded 0.

## 3. Methods and Results

### 3.1. Statistical Analysis

Data were processed with SPSS 13.0 software (SPSS Inc., Chicago, IL, USA). Two-sided test was applied. When inspection level *α* = 0.05, *p* < 0.05 is considered statistically significant. Enumeration data is described by frequency. The correlation between the categorical independent variables (four properties, five flavors, and channel tropism) and the dependent variable (hepatotoxicity and nonhepatotoxicity of Chinese HMs) was analyzed by chi-square test for two-way unordered categorical data. Unconditional LR was applied to screen the influential factors, and the LR prediction model was established. The goodness-of-fit of the LR was evaluated with Hosmer-Lemeshow test and statistical significance of the model was tested with chi-square test.

SPSS software package was used to draw the receiver operating characteristics (ROC) curve and then to calculate the area of under the ROC curve (AUC). The predictive power was evaluated. Besides, sensitivity, specificity, and overall accuracy were calculated, and the accuracy of the prediction model was evaluated.

### 3.2. Screening the Input Variable for the Prediction Model

#### 3.2.1. The Correlation of Hepatotoxic and Nonhepatotoxic Chinese HMs with Four Properties Analyzed with Chi-Square Test for Two-Way Unordered Categorical Data

**Figure 1:**
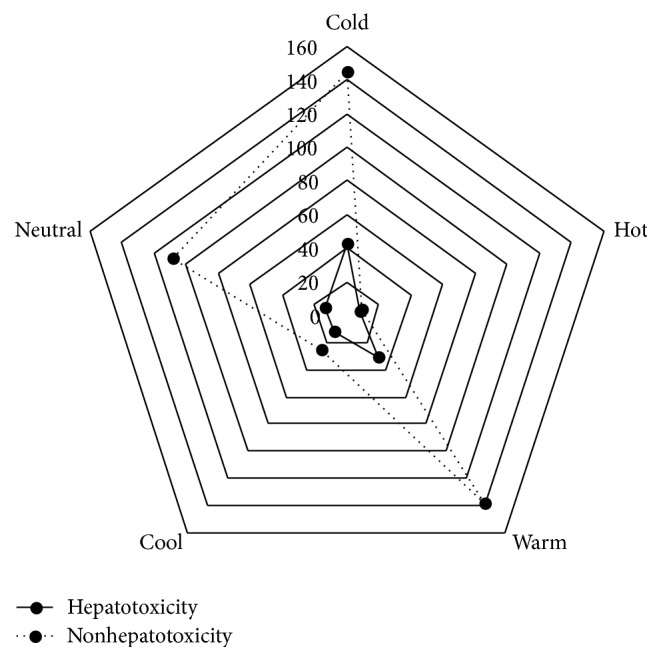
Hepatotoxicity and nonhepatotoxicity of the frequency distribution in four properties: *χ* ^2^ = 17.52 and *p* = 0.002.

In Figure 1, the frequency counts of 107 hepatotoxic and 431 nonhepatotoxic HMs in four properties is shown. The data were processed with chi-square test for two-way unordered categorical data. According to the results, hepatotoxic and nonhepatotoxic HMs are correlated with four properties (*p* < 0.05), and the coefficient is 0.178. Although the result is of statistical significance, the value is low. Therefore, it can be concluded that there is a weak correlation of hepatotoxic and nonhepatotoxic Chinese HMs with four properties.

#### 3.2.2. The Correlation of Hepatotoxic and Nonhepatotoxic Chinese HMs with Five Flavors Analyzed with Chi-Square Test for Two-Way Unordered Categorical Data

**Figure 2:**
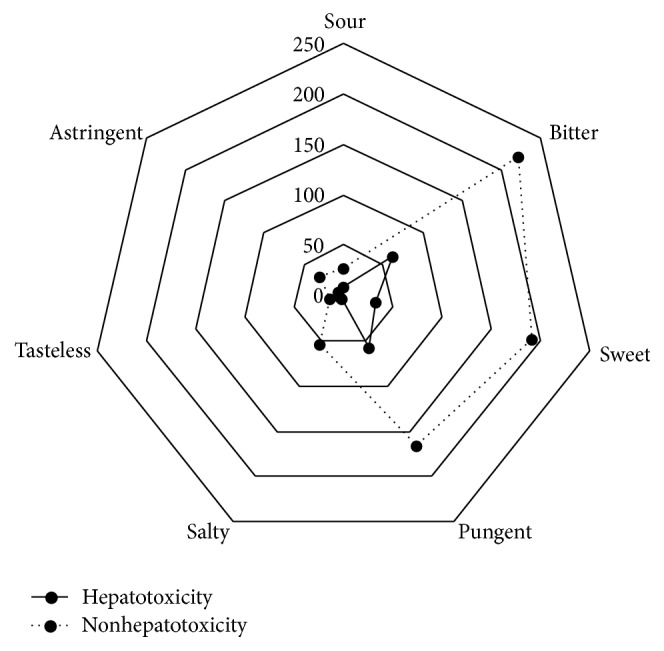
Hepatotoxicity and nonhepatotoxicity of the frequency distribution in five flavors: *χ* ^2^ = 18.81, *p* = 0.004.

In Figure 2, the frequency count of 107 hepatotoxic and 431 nonhepatotoxic Chinese HMs in five flavors is shown. The data were processed with chi-square test for two-way unordered categorical data. According to the results, hepatotoxic and nonhepatotoxic Chinese HMs are correlated with five flavors (*p* < 0.05), and the coefficient is 0.145. Although the result is of statistical significance, the value is low. Therefore, it can be concluded that there is a weak correlation of hepatotoxic and nonhepatotoxic Chinese HMs with five flavors.

#### 3.2.3. The Correlation of Hepatotoxic and Nonhepatotoxic HMs with Channel Tropism Analyzed with Chi-Square Test for Two-Way Unordered Categorical Data

In Table 1, the frequency count of 107 hepatotoxic and 431 nonhepatotoxic HMs in channel tropism is shown. The data were processed with chi-square test for two-way unordered categorical data. There are 6 table cells with theoretical frequency less than 5, accounting for 1/4 of the total table cells. Generally, in a row × column table, the theoretical frequency in each cell should not be less than 1, and cells with the theoretical frequency ≥1 and ≤5 should not exceed 1/5 of the total table cells. Otherwise, the following method should be applied: increasing the sample size to increase the theoretical frequency, eliminating the row or column with theoretical frequency that is too low, or combining such row or column with a neighboring one, based on the specific knowledge for the research; instead, Fisher's exact test on two-way unordered row × column contingency table was applied [18]. According to the TCM theory, heart meridian and small intestine meridian are correlated from the external to the internal, and so are pericardium meridian and triple energizer meridian [19]; and therefore, based on this understanding or specific knowledge, data of the heart meridian and small intestine meridian were combined together and so were the data of the pericardium meridian and tripe energizer meridian (Table 2). After being processed with chi-square test for two-way unordered categorical data, it is shown that there is no correlation of hepatotoxic and nonhepatotoxic Chinese HMs with channel tropism (*p* = 0.71), and the coefficient is 0.068. Therefore, it can be concluded that there is no correlation of hepatotoxic and nonhepatotoxic Chinese HMs with channel tropism.

From the above analysis, it could be concluded that the hepatotoxic and nonhepatotoxic Chinese HMs are correlated with four properties and five flavors but are not correlated with channel tropism. Hence, four properties (cold, hot, warm, cool, and neutral) and five flavors (sour, bitter, sweet, pungent, salty, tasteless, and astringent) were the variables to be input for the prediction model.

### 3.3. The Design of LR Prediction Model

#### 3.3.1. LR Model

LR is widely used in the medical field [20], and it can be binomial or multinomial. Binomial or binary LR deals with some situations in which the observed outcome for a dependent variable only has two possible types. Multinomial LR deals with situations where the outcome can have three or more possible types. In this research, binomial LR was applied to predict the hepatotoxicity of Chinese HMs.

#### 3.3.2. Unconditional LR and Setup of the LR Prediction Model

After being processed with the chi-square test for the two-way unordered categorical data, it is shown that the hepatotoxic and nonhepatotoxic Chinese HMs are correlated with four properties and five flavors. For the LR, whether the HMs are hepatotoxic is the dependent variable; four properties (cold, hot, warm, cool, and neutral) and five flavors (sour, bitter, sweet, pungent, salty, tasteless, and astringent) are the independent variables. The forward stepwise selection was used to screen the significant variables. According to the results, there are four variables selected for LR model, that is, pungent, salty, warm, and neutral, with the odds ratio (OR) (95% confidence interval (CI)) being 2.04 (1.26, 3.292), 0.26 (0.08, 0.857), 0.48 (0.28, 0.83), and 0.38 (0.20, 0.72), respectively (Table 3). Pungent is the risk factor, while salty, warm, and neutral are the protective factors.

LR model is as follows:

(1) $$\begin{array}{c} p=\frac{1}{1+\exp\left(1.231-0.712*pungent+1.354*salty+0.726*warm+0.971*neutral\right)}. \end{array}$$

Chi-square test was applied to test the significance of this LR prediction model (*χ* ^2^ = 37.006, *p* = 0.000). According to the results, the variables to be input in the prediction model for hepatotoxic and nonhepatotoxic Chinese HMs showed predictive power with obvious significance, and the goodness-of-fit of the prediction model was evaluated by Hosmer-Lemeshow test, showing that the overall model fit is good (*χ* ^2^ = 4.355, df = 8, and *p* = 0.824).

**Figure 3:**
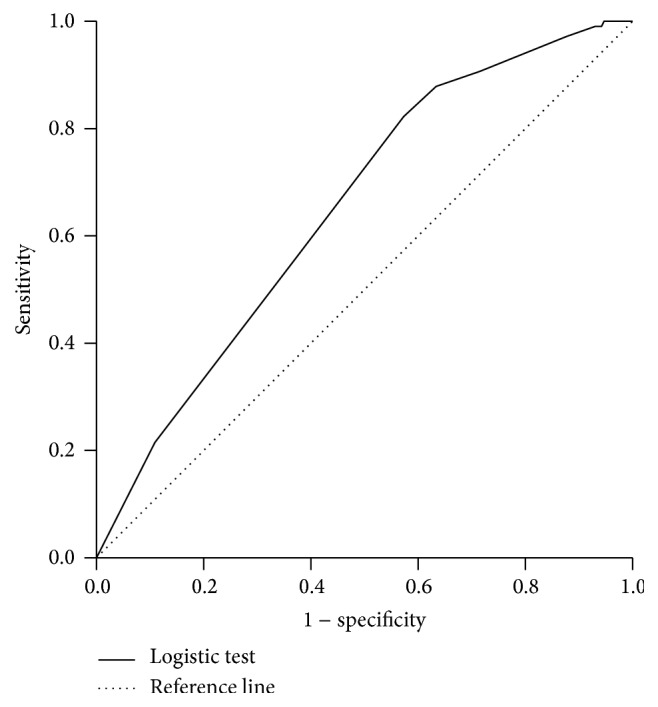
The ROC curve of logistic regression to predict the probability.

The ROC curve was drawn with the sensitivity as the vertical coordinates, and 1 − specificity as the horizontal coordinates (Figure 3).

In the ROC curve analysis, the sensitivity and specificity are well combined. ROC curve analysis is used for quantitative analysis based on the area under the curve, and the results are not affected by positive rate. One of the most popular measures of the accuracy of a diagnostic test is the AUC. The AUC can take on values between 0.0 and 1.0. A test with an AUC 1.0 is perfectly accurate. Diagnostic tests with an AUC greater than 0.5 have certain predictive ability. The cutoff value is 0.5 [21]. Therefore, ROC curve analysis could also be used for evaluating the LR. In this research, AUC is 0.65 for this LR prediction model and is of obvious significance compared with AUC of 0.5 (*p* < 0.01), with 95% CI being 0.596–0.704, which means relatively weak predictive power. The optimal cutoff value (*p* = 0.204) was determined by calculating the point on the ROC curve with the maximum Youden index (sensitivity − [1 − specificity]) (Figure 3).

### 3.4. Analysis of Prediction Result

The data were put into the prediction model to get the prediction probability. When the cutoff value for the prediction probability was 0.204, in the 431 hepatotoxic Chinese HMs, 184 nonhepatotoxic Chinese HMs were misjudged as hepatotoxic one and, in 107 hepatotoxic Chinese HMs, 19 hepatotoxic were misjudged as nonhepatotoxic Chinese HMs. The overall accuracy rate was 50.558% (Table 4).

## 4. Discussion

This research aims to establish an LR prediction model for hepatotoxicity of Chinese HMs based on TCM theory and to provide a statistical basis for predicting hepatotoxicity of HMs.

### 4.1. Analysis on Influential Factors Affecting Hepatotoxicity of Chinese HMs

The correlation between the independent variables (four properties, five flavors, and channel tropism) and the dependent variable (hepatotoxicity and nonhepatotoxicity of Chinese HMs) was evaluated with chi-square test for two-way unordered categorical data, and the result showed that hepatotoxic and nonhepatotoxic Chinese HMs had no correlation with the channel tropism but had correlation with four properties and five flavors. Unconditional LR was applied to screen the influential factors from the two categories of variables, totally 12 variables. The influential factors include warm and neutral from four properties and pungent and salty from five flavors. Pungent is the risk factor while salty, warm, and neutral are the protective factors for hepatotoxicity of Chinese HMs.

Hepatotoxic and nonhepatotoxic Chinese HMs have no obvious difference in the distribution on channel tropism. The so-called hepatotoxic Chinese HMs by biomedicine actually have multiple channel tropism, and the so-called nonhepatotoxic Chinese HMs have multiple channel tropism, too. This might be due to the fact that the organs in biomedicine do not correspond with the organs in TCM [22]. For example, the liver in biomedicine is different from that in the TCM. Channel tropism means that Chinese HMs could selectively work on organs or channels in TCM but does not mean that they could work on the organs of biomedicine. Therefore, this difference in the concept of organs might be the cause that channel tropism is not the factor to affect the hepatotoxicity of Chinese HMs.

It is generally held that the volatile oil, glycosides, and alkaloids are the main material basis of pungent HMs [23]. Research on hepatotoxic components of Chinese HMs in the last 10 years has shown that the volatile oil, glycosides, and alkaloids possibly are the material basis for hepatotoxicity of most Chinese HMs [24], which might be the possible reason that pungent is the risk factors for the hepatotoxicity of Chinese HMs.

According to the TCM theory, salty flavor has the function to soften the hard mass and cause purgation. Modern pharmacological research has shown that inorganic salts and iodine are the main components of salty Chinese HMs [23], and they both might reduce the reabsorption of water and other substances by the intestinal tract. Research has shown that a delayed onset of acute liver failure might be related to toxic substances circulating in the intestines and liver for long time [25]. Therefore, it could be concluded that salty Chinese HMs could reduce hepatotoxicity of Chinese HMs by preventing the absorption of toxic substance in the intestinal tract and thus reduce circulation of toxic substance in the intestines and liver, which could be a possible reason that salty flavor is a protective factor for hepatotoxicity of HMs.

TCM holds that four properties are the summarization of the body reactions due to the action by medicine. Chinese HMs with the function to promote body to become warm or hot are the warm or hot Chinese HMs (hot is stronger than warm); otherwise, the Chinese HMs are cold or cool. The neutral is the one neither hot nor cold. In this study, the hepatotoxic or nonhepatotoxic Chinese HMs of hot properties are less than those of cold, cool, warm, and neutral properties, which might be the possible reason that hot property did not show correlation with hepatotoxicity of Chinese HMs according to our statistical analysis.

Research has shown that, in the experimental rats with cold patterns, Chinese HMs with warm and hot properties could promote the metabolism of glucose and lipid and protein synthesis in the mitochondria, therefore to increase the energy metabolism in the mitochondria [26]. Another research has shown that* táo rén* (*Semen Persicae*) with neutral properties has the function to promote or suppress the cyclic AMP-dependent protein kinase (PKA) cAMP/PKA signal pathway in the experimental rats with blood stasis of cold or warm patterns [27]. Also other researches present evidence that mitochondria are sensitive to the level of cAMP/PKA signaling and can respond by modulating levels of respiratory activity or committing to self-execution, and inhibition of the *β*-adrenergic receptor/cAMP/PKA axis protects against the oxidant-mediated cell injury [28, 29]. Besides, other researches have shown that benzbromarone could decrease the mitochondrial membrane potential, reduce the enzyme activities in the mitochondria, induce mitochondrial uncoupling, and decrease the adenosine triphosphate (ATP) production, therefore to cause cell apoptosis and necrosis [30, 31]. Some ingredients of Chinese HMs also could induce mitochondrial apoptosis, which could be the underlying mechanism for hepatotoxicity [32]. Some researchers have shown that the adiponectin could alleviate acetaminophen-induced hepatotoxicity by promoting and mediating autophagy of damaged mitochondria [33]. Therefore, it could be concluded that the protective mechanism of Chinese HMs with warm properties is possibly through enhancing mitochondrial function, accelerating biological oxidation in cells, increasing basal metabolic rate, and promoting excretion of toxic metabolites, while Chinese HMs of neutral property have the protective functions possibly through inhibiting the absorption of toxic substances in the early stage of liver damage and enhancing the excretion of toxic substances in the later stage.

### 4.2. The Significance of LR Prediction Model on Research of Hepatotoxicity of Chinese HMs

In the analysis on the LR prediction model of hepatotoxicity of Chinese HMs, we have established that, in the 431 nonhepatotoxic Chinese HMs, 184 were misjudged as hepatotoxic; in the 107 hepatotoxic Chinese HMs, 19 were misjudged as nonhepatotoxic. The misjudgment rate was 49.44%. The possible reasons are as follows: the inclusion criterion for the hepatotoxicity is so broad that the included variables from the four properties and five flavors show weak correlation with the hepatotoxic Chinese HMs.

Research has also shown that the hepatotoxicity could be further classified. For example, HMs with the cold property show an intensive effect in liver hepatitis, HMs with the hot property show a stronger effect in liver cirrhosis, and both of them manifest effects in hepatocellular carcinoma [34]. Therefore, if the inclusion criterion for the hepatotoxicity could be further subdivided with more categories, the included variables from the four properties and five flavors could show strong correlation with the hepatotoxicity of different categories, the misjudgment rate will become low, and the predictive power of the model will increase.

In the analysis on the misjudgment of this LR prediction model for hepatotoxicity, it is shown that the ratio for nonhepatotoxic Chinese HMs being misjudged as hepatotoxic is 43.38%, and the ratio for hepatotoxic Chinese HMs being misjudged as nonhepatotoxic is 17.75%. Therefore, it could be concluded that the judgment for hepatotoxicity of Chinese HMs is stricter than that for nonhepatotoxicity of Chinese HMs by using this prediction model. If this model is applied to the development of new drugs from Chinese HMs, it will be more likely to avoid the risk of hepatotoxicity and to ensure the safety of Chinese HMs. Besides, researches have shown that various HMs which are traditionally considered “nontoxic” are gradually found to have potential hepatotoxicity [4, 5]. Therefore, there is the possibility that the “nonhepatotoxic Chinese HMs” misjudged as hepatotoxic in this prediction model could be hepatotoxic, which should be verified in the future research.

In the future, with the development of the research of hepatotoxicity of Chinese HMs, there will be more and more data available for the establishment of the prediction model, and the predictive power of this logistic regression prediction model for hepatotoxicity will be increased correspondingly.

## 5. Conclusions

In this research, four properties, five flavors, and channel tropism, 24 variables from 3 categories, were analyzed for their correlations with the hepatotoxicity of Chinese HMs. There were 12 variables from four properties and five flavors to be input for LR and to establish an LR prediction model. This research employs a new method by combing TCM theory with modern statistics. By combining TCM theory with modern research on hepatotoxicity, this research provides a prediction model for hepatotoxicity of Chinese HMs based on statistics. The method employed in this research is promising for predicting hepatotoxicity of Chinese HMs and could provide some references for avoiding hepatotoxicity in the development of new drugs from Chinese HMs. The combination of generality and vagueness of TCM theory and accuracy of modern science could be a new method for combing biomedicine with TCM.

## Figures and Tables

**Table 1:** Chinese HMs in channel tropism by frequency count (original data).

Classification	Channel tropism (meridian)												
	Lung	Large intestine	Stomach	Spleen	Heart	Small intestine	Bladder	Kidney	Pericardium	Triple energizer	Gall bladder	Liver	Total
Hepatotoxicity	36	23	34	35	18	3	9	27	1	1	5	71	263
Nonhepatotoxicity	174	77	146	140	102	12	46	126	3	1	18	244	1089

Total	210	100	180	175	120	15	55	153	4	2	23	315	1352

**Table 2:** Chinese HMs in channel tropism by frequency statistics (combined data).

Classification	Channel tropism (meridian)										
	Lung	Large intestine	Stomach	Spleen	Heart and small intestine	Bladder	Kidney	Pericardium and triple energizer	Gall bladder	Liver	Total
Hepatotoxicity	36	23	34	35	21	9	27	2	5	71	263
Nonhepatotoxicity	174	77	146	140	114	46	126	4	18	244	1089

Total	210	100	180	175	135	55	153	6	23	315	1352

*χ* ^2^ = 6.28 and *p* = 0.71.

**Table 3:** Variables in the equation.

	*p*	OR	95% CI	
			Lower	Upper
Pungent	0.004	2.037	1.261	3.292
Salty	0.027	0.258	0.078	0.857
Warm	0.008	0.484	0.283	0.828
Neutral	0.003	0.379	0.201	0.715
Constant	0.000	0.292		

**Table 4:** Classification table.

Observed	Predicted		Accuracy (%)
	Nonhepatotoxicity	Hepatotoxicity	
Nonhepatotoxicity	184	247	42.691
Hepatotoxicity	19	88	82.243
Overall percentage			50.558

The cutoff value is 0.204.
